# Translation and Validation Study of the French Version of the eHealth Literacy Scale: Web-Based Survey on a Student Population

**DOI:** 10.2196/36777

**Published:** 2022-08-31

**Authors:** Noémie Chaniaud, Camille Sagnier, Emilie Loup-Escande

**Affiliations:** 1 Centre de Recherche en Psychologie : Cognition, Psychisme, Organisations (UR 7273) Université de Picardie Jules Verne Amiens France

**Keywords:** eHealth Literacy Scale, eHEALS, eHealth literacy, transcultural validation process, Health Literacy Survey–Europe, HLS-EU

## Abstract

**Background:**

eHealth literacy is emerging as a crucial concept for promoting patient self-management in an overloaded hospital system. However, to the best of our knowledge, no tool currently exists to measure the level of eHealth literacy among French-speaking people. The eHealth Literacy Scale (eHEALS) is an easy-to-administer 8-item questionnaire (5-point Likert scale, *ranging from strongly disagree to strongly agree*) that has already been translated into many languages. Currently, it is the most cited questionnaire in the literature.

**Objective:**

The aim of this study was to translate eHEALS to French and validate the French version of eHEALS (F-eHEALS).

**Methods:**

The validation of the F-eHEALS scale followed the 5 steps of the transcultural validation method: double reverse translation, validation by a committee of experts (n=4), pretest measurement to check the clarity of the items (n=22), administration of the scale in French via a web-based quantitative study combined with two other questionnaires (Health Literacy Survey-Europe–16 and Patient Activation Measure–13; N=328 students), and finally test-retest (n=78) to check the temporal stability of the measurements obtained from the scale.

**Results:**

The results obtained for the measurement of factor structure, internal consistency, and temporal stability (intraclass correlation coefficient=0.84; 95% CI 0.76-0.9; *F*_77,77_=6.416; *P*<.001) prove the validity and fidelity of the proposed scale. The internal consistency of F-eHEALS was estimated by Cronbach α of .89. The factor analysis with varimax rotation used to validate the construct showed a 2-factor scale. The effect of the construct was analyzed using 3 hypotheses related to the theory. The F-eHEALS score was correlated with the Health Literacy Survey-Europe–16 score (*r*=0.34; *P*<.001) and the Patient Activation Measure–13 score (*r*=0.31; *P*<.001).

**Conclusions:**

F-eHEALS is consistent with the original version. It presents adequate levels of validity and fidelity. This 2D scale will need to be generalized to other populations in a French-speaking context. Finally, a version taking into account collaborative applications (ie, Health 2.0; eg, Digital Health Literacy Instrument scale) should be considered on the basis of this study.

## Introduction

### Background

eHealth (or connected health) is an emerging field that incorporates various stakeholders, for instance, in the fields of medical informatics, public health, and companies [1]. This field can be an interesting opportunity to overcome the weaknesses of the current health systems and help health professionals and patients by making them active in their own health [1,2]. To benefit from this emerging field, connected medical devices available to the general public have to be accessible to all user profiles [3,4], regardless of the environment in which they are used. This means that patients must be *able* to use these tools correctly [5]. Using health information technology requires a specific set of knowledge and skills such as the ability to read, use computers, search for information, understand health information, and contextualize it [6]. All these skills relate to *eHealth literacy* [6]. In other words, assessing individuals’ skills in using eHealth is equivalent to assessing their level of eHealth literacy. eHealth literacy scales have been developed to address this need [7].

### eHealth Literacy: Definition and Theoretical Models

According to the *Institute of Medicine*, eHealth literacy refers to a person’s skills “to search for, find, understand and evaluate health information from electronic sources and to apply the knowledge gained to treat or solve a health problem” [8]. This definition highlights the importance of contextual factors, including the media through which health information is disseminated and the level of health literacy in relation to these media [6]. Current media for the diffusion of health information include interactive tools for behavior change, such as applications, websites, and phone support services [9,10]. However, there is significant difference between the use of technology and the use of web-based health information (ie, digital literacy or eHealth literacy) [11], even if both the use of technology and the use of web-based health information are activities associated with eHealth literacy. Therefore, Norman and Skinner [6] aimed to identify the skills required to use this information.

Early studies in this field focused on *general* literacy (ie, “the ability to understand and use written information...to achieve personal goals and expand one’s knowledge and abilities” (p12) [12]. Research has expanded into areas such as health literacy and eHealth literacy in relation to patient health [13]. The fundamental theories of eHealth literacy are, in part, based on social cognitive theories [14] and self-efficacy theories [15-17]. These theories consider self-confidence as a precursor of the behavior changes and skills needed to acquire high level of eHealth literacy. On the basis of these theories, Norman and Skinner [6] proposed a model of eHealth literacy (*lily model*) based on six different skills (or *literacies*) applied to health: (1) *traditional* literacy and numeracy, (2) health literacy, (3) information literacy, (4) scientific literacy, (5) media literacy, and (6) computer literacy. According to Norman and Skinner [6], eHealth literacy consists of a combination of all these 6 *core literacies*. These authors developed the *eHealth Literacy Scale* (eHEALS), an eHealth literacy rating scale to promote eHealth and identify strategies to help patients use digital media in health. The eHEALS does not measure skills directly, but “measures consumers’ *perceived* skills and comfort with eHealth” (p24) [6].

### eHealth Literacy Scales and Cross-cultural Validations

Currently, several tools are available to assess eHealth literacy. In a systematic review of health literacy instruments [18], the authors identified 8 instruments including the eHEALS [7], *eHealth Literacy Questionnaire* [19], *eHealth Literacy Framework* [20], *Digital Health Literacy Instrument* (DHLI) [21], *eHealth Literacy Assessment Toolkit* [22], eHEALS-Extended [23], Electronic Health Literacy Scale [24], and *Transactional eHealth Literacy Instrument* [25]. To the best of our knowledge, none of these scales have been validated in French.

Several reasons led us to choose to validate eHEALS in French [7]. First, the eHEALS is a relatively short tool (8 items), making it easy to administer and combine with other scales. Second, currently, it is the most widely used scale for measuring eHealth literacy in international scientific literature [26]. This tool has been translated into different languages over the past 10 years [27-39]. This shows that it can be used in various languages and cultural contexts. However, currently, its French translation does not exist. Furthermore, one of the advantages of eHEALS is that it can be adapted to different populations and different contexts. The scale was administered to different categories of individuals, such as older people [40], young adults [41], nursing students [42], and teenagers [43].

### The eHEALS Original Version

The original version of eHEALS, created by Norman and Skinner [7], is composed of 10 items relating to the 6 literacy types of the *lily* model mentioned previously. A total of 8 items assess users’ knowledge; comfort; and perceived skills in finding, assessing, and applying digital health information to answer health questions. The scale also includes 2 additional items that focus on participants’ perception of the use of the internet as a decision-making tool and its usefulness in collecting health information (these items are not included in the total score).

The psychometric characteristics of the original scale were assessed on a sample of teenagers (N=664; mean age 14.95, SD 1.24 years). Cronbach α was .88, and the test-retest reliability was 0.68 [7]. The authors used factor analysis and highlighted a single-factor solution (eigenvalue=4.479; 56% of the variance explained).

### Factors Affecting eHealth Literacy

Several factors are associated with eHealth literacy: sociodemographic characteristics, health literacy, and commitment to health. Studies do not agree on the association with sociodemographic characteristics (gender, education level, and health outcomes) [38,44-46]. As some studies show differences in eHealth literacy according to these sociodemographic characteristics and others do not, obtaining an overview of the eHealth literacy level of a population is complex. Chesser et al [47] showed in a systematic review that eHealth literacy was associated with the level of education and advanced age, even if there is some variability among the older population.

Contrary to the association with sociodemographic characteristics, there seems to be a consensus on the relationship between health literacy and eHealth literacy [38,45,48,49], health literacy being one of the components of eHealth literacy in the model suggested by Norman and Skinner [6]. Neter et al [49] found a positive, moderate, and significant correlation between the scores on Health Literacy Survey-Europe–16 (HLS-EU–Q16) and eHEALS (*r*=0.36; *P*<.05) in a sample of 199 adults. Similar results (*r*=0.43; *P*<.001) were obtained by Duplaga et al [48] on a sample of 199 young adults (aged 18-29 years). Wångdahl et al [38] also found a moderate positive correlation (*r*=0.47; *P*<.05) between the HLS-EU–Q16 and the Swedish version of eHEALS in a sample of 323 adults.

Furthermore, there seems to be a link between patient commitment to health and the level of eHealth literacy. Patient commitment to health, also known as *patient activation*, is commonly measured by the *Patient Activation Measure–13* (PAM-13) scale [50]. For instance, Lee et al [51] assessed the level of activation among 399 adults who were chronically ill, using PAM-13 and their eHealth literacy level using eHEALS. The authors showed a positive, moderate, and significant correlation between these 2 variables (*r*=0.50; *P*<.001).

In summary, previous studies agree on the link between health literacy and eHealth literacy and the link between patient health activation and eHealth literacy. However, the links between the sociodemographic characteristics of individuals and eHealth literacy seem to be less convergent.

### Objective

The objective of this study was to translate eHEALS, which is already developed by Norman and Skinner [7], into French and validate it with a student population.

## Methods

### Procedure

To conduct the translation, adaptation, and validation of eHEALS [7], we followed the American Psychological Association guidelines [52] and the 5 steps recommended by Vallerand [53]: translation, validation with experts, pretest measurement, administration of the tool, and test-retesting. Figure S1 in Multimedia Appendix 1, translated from Vallerand [53], shows these steps.

These 5 steps were conducted between December 2019 and March 2020.

### Ethics Approval

This study complied with the principles set out in the Declaration of Helsinki of 1964 and its subsequent amendments. Before the experiment, the participants signed a web-based consent form, and the questionnaire was validated by the research ethics committee of the university Picardie Jules Verne. The participants did not receive any financial compensation for their participation, and they agreed to participate in the study. The anonymity, confidentiality, and secure storage of the data were guaranteed to the participants and respected.

### Step 1: Translation of eHEALS Into French by a Process of Double Reverse Translation—Preparation of a Preliminary Version

We have been authorized to translate the original eHEALS scale by its authors [7]. After authorization, we performed a double reverse translation of the eHEALS by 2 professional translators who had French as their native language, independently of each other. This method is considered “ideal for drafting the psychological instrument” (our translation; p665) [53]. Then, the 2 French versions obtained were retranslated into English by 2 professional translators who had English as their native language and had not seen the original version, again independently of each other. In total, 2 French versions and 2 preliminary English versions were produced at the end of this phase.

### Step 2: Validation by a Committee of Experts

A committee of 4 experts, 3 (75%) of whom are authors of the manuscript, consisting of 1 (25%) expert in neuropsychology, 1 (25%) expert in cognitive psychology, and 2 (50%) other experts in ergonomics, met to examine the quality of the translations and agree on the best version. To do so, they compared the different translated versions with the original English version, taking into account the French cultural context and checking the clarity of the language. This committee made some minor changes to the selected items. A French version of eHEALS (F-eHEALS) was established after the committee’s intervention. The committee members discussed in detail the translation of the term *health resources*. The suggestions for translation were as follows: “ressources en santé,” “ressources sur la santé,” or “ressources de santé.” Members agreed on “ressources sur la santé” as being more inclusive and easy to understand. The committee was also unsure whether to propose the term “information” instead of “resources.” Finally, the term “resources” (“ressources” in French) was retained to not differ from the original version.

### Step 3: Pretest Measurement for Clarity of Items

We performed a pretest measurement to check the clarity of the items (ie, unambiguous wording of the translated items). A total of 22 participants were asked to evaluate the items (including the instructions) using a web-based questionnaire. To do so, participants had to read each item and judge its clarity on a scale from 1 (*not at all clear)* to 7 (*very clear)*. Items that were scored ≤4 needed to be reviewed. For each item, participants could also leave comments on potential ambiguities and justify their scores for each item. The results of this pretest measurement were used to create a final version of F-eHEALS.

### Step 4: Administration

A total of 344 students aged 16 to 33 years responded to F-eHEALS, two additional scales (HLS-EU–Q16 and the PAM-13), and questions on their sociodemographic characteristics.

This population was relevant because the suitability of the tool for a population of young adults has already been demonstrated [32,33,35,37,41,42]. To target this population, the scales were shared on Facebook groups of students from several French universities. All respondents had to be students and have French as their native language.

The instructions given to the participants explained the main objective of the study regarding the translation of the eHEALS scale and provided some explanations to justify the presence of the other questionnaires (ie, HLS-EU–Q16 and PAM-13): “It concerns your perceptions of your ability to search, find and process health-related information on the Internet, your involvement in your health.” As there appears to be a relationship among patient commitment, health literacy, and eHealth literacy, we chose to add PAM-13 and HLS-EU–Q16 to the questionnaires to measure concurrent validity.

Each participant had to consent to the study by means of electronic validation to be able to access the questionnaires. First, the participants completed information about their age, gender, level of education, and field of education and a question related to their health conditions. Then, they were asked to complete F-eHEALS, HLS-EU–Q16 [54], and finally, PAM-13 [50]. After completing the questionnaires, participants were asked if they wanted to be contacted again for the test-retest by providing an email address.

### Step 5: Test-Retest

Test-retest stability is the best indicator of the metric quality of a scale relative to other fidelity indices [55]. This evaluation has the specificity of measuring the temporal stability of the measurements [53]. A total of 170 participants agreed to be contacted on a later date. The same questionnaires with the same format (instructions, consent, and questionnaires) were sent to the participants 1 month after their initial enrollment. Of the 170 participants, 84 (49.4%) participants responded to the questionnaires.

### Measures: Questionnaires and Data Analysis

#### F-eHEALS Questionnaire

F-eHEALS, similar to the original version of eHEALS developed by Norman and Skinner [7], consists of 8 items measuring eHealth literacy on a 5-point Likert scale (ranging from 1=*strongly disagree* to 5=*strongly agree*). A total of 2 other items, related to the importance and usefulness that individuals attach to the internet for making decisions about their health, are included, but are not to be counted in the final rating. The eHEALS score depends on the points obtained for each item (*strongly disagree* scores 1 point and *strongly agree* scores 5 points). The eHEALS score ranges from 8 to 40 points. The higher the score, the higher the level of eHealth literacy. The analysis of the items was conducted on the 8 items that make up eHEALS [7].

#### HLS-EU–Q16 Scale

The *HLS-EU–Q16* [54] is the short version of *HLS* developed by Sørensen et al [56]. It has been translated into French [57]. This version consists of 16 items, 13 (81%) of which assess the 4 types of health literacy skills: the ability to access, understand, evaluate, and apply health information [56]. The respondent has to assess their own ability to access the information (eg, “Please indicate on a scale from very easy to very difficult, how easy it is for you to understand your doctor’s or pharmacist’s instructions on how to take your medication?”). Overall, 4 categories of answers were proposed on a 4-point Likert scale. *Difficult* or *very difficult* responses do not score any points, whereas *easy* and *very easy* responses score 1 point. Then, the total score is calculated: the higher the score, the higher the level of health literacy.

#### French Version of PAM

PAM-13 [50], translated into French [58], is a 13-item scale that assesses a patient’s knowledge, skills, and confidence in self-managing their health or chronic illness. The respondent has to assess their ability to self-manage their health (eg, “All things considered, I am the person who is responsible for taking care of my health”). Respondents provide their answers on a 5-point Likert scale (from 1=*strongly disagree* to 5=*strongly agree*). Then, the total score is calculated based on the participants’ response to each item. The total score for the items ranges from 13 to 65 points; the higher the score, the higher the level of commitment to health.

### Data Analysis

#### Overview

The results were analyzed using SPSS (version 22; IBM Corp). All the data of this study are in open access [59]. The fidelity assessment was performed by analyzing the internal consistency of the tool, as assessed by Cronbach α. Cronbach α was >.7, which indicates that the items in the study are consistent [60]. Construct validity was measured by means of three statistical analyses: (1) exploratory factor analysis (principal component analysis with varimax rotation), (2) analysis of interitem correlations, and (3) analysis of construct effects using Pearson correlations among HLS-EU–Q16, PAM-13, and F-eHEALS.

#### Exploratory Factor Analysis

To verify whether the measures are suitable for factor analysis, we used the Kaiser-Meyer-Olkin (KMO) and Bartlett sphericity tests. KMO values close to 1 are considered to be ideal, and statistically significant result in the Bartlett sphericity test shows that the correlation matrix is not an identity matrix. The multivariate normality test (distance of Mahalanobis) was used to ensure the normality of the data. If the Mahalanobis maximum value is less than the critical value, multivariate normality is existing. Then, we used principal component analysis (varimax rotation; Kaiser criterion >1), which is a multivariate interdependence technique that allows the associated variables and the measurement of latent constructs to be determined. To conduct this analysis, a minimum statistical power is required. Hair et al [61] consider it necessary to have a ratio of 10 participants per variable in the analysis, which will correspond to a minimum of 80 participants for our scale. Factor scores >0.71 were considered to be excellent, those >0.63 to be very good, those >0.55 to be good, those >0.45 to be acceptable, those close to 0.32 to be poor, and those <0.32 to be very poor [62].

#### Analysis of Interitem Correlations

The analysis of interitem correlations allows the internal coherence of the scale to be assessed. To measure this construct, the elements must be sufficiently correlated (*r*>0.4).

#### Analysis of the Effects of Constructions

The construct effect makes it possible to verify the links between the construct and the variables identified in the literature [53]. We formulated 3 hypotheses about the links between health literacy and other variables that have previously been shown to be related to eHealth literacy. The measurement of the effect of the construction is based on 3 hypotheses: (1) the level of eHealth literacy is not correlated with sociodemographic characteristics (gender and health outcomes), (2) the level of eHealth literacy will be positively and moderately correlated with the level of health literacy, and (3) the level of eHealth literacy will be positively and moderately correlated with the level of patient activation. We used Pearson correlations to validate (or invalidate) the hypotheses.

## Results

### Translation and Equivalence Verification With the Original Version (Steps 1, 2, and 3)

During the pretest measurement to assess item clarity (n=22; mean age 38.47, SD 8.44 years; range 26-63 years), none of the F-eHEALS items were rated <4 (Multimedia Appendix 2). All items had been rated a mean score of 6.27 (SD 1.15) and, therefore, were considered to be understandable and clear. Only items 5 and 6 were modified in a minor way by changing the term “ressources” to “informations,” the first term being considered to be very confusing by the respondents. This term had already been discussed by the expert committee.

### Validation (Steps 4 and 5)

#### Sociodemographic Characteristics of the Sample

Of the 344 participants who responded to this scale, we excluded 15 (4.4%) participants: 7 (47%) participants were not students, 8 (53%) were aged >35 years and thus considered to cause risk of age bias, and 1 (7%) did not give their consent. Thus, 95.3% (328/344) of the participants (mean age 21.22, SD 2.7 years; 274/328, 83.5% were women; 52/328, 15.9% were men; and 2/328, 0.6% were nonbinary) were eligible for step 4. Details of respondent characteristics are presented in Multimedia Appendix 3.

#### Internal Consistency and Temporal Stability

Of the 344 participants who responded to the questionnaires, 170 (49.4%) participants agreed to be recontacted for the retest. Of these 170 participants, 84 (49.4%) responded to the questionnaire. Of the 84 participants, 6 (7%) participants had to be excluded because their identification did not allow a link to be made with the database of contacts from the first test. Thus, 93% (78/84) of the participants were included in the retest. The temporal stability of our sample was (intraclass correlation coefficient=0.84; 95% CI 0.76-0.9; *F*_77,77_=6.416; *P*<.001). We also observed a strong and positive correlation between the 2 sessions (*r*=0.72; *P*<.001). The internal consistency of F-eHEALS was estimated by a Cronbach α of .89.

### Assessment of Construct Validity

#### Exploratory Factor Analysis by Principal Component Analysis

The Bartlett sphericity test was significant (N=328; *χ*^2^_28_=1616.3; *P*<.001), and the KMO index was 0.85. The multivariate normality test was performed (distance of Mahalanobis: dof=8; mean 7.976, SD 5.317; minimum=0.876; maximum=30.146). None of the outliers were removed.

The analysis of the main factor produced an eigenvalue of 4479. The first 2 factors were extracted on the basis of Kaiser criteria, because they have an eigenvalue >1. The first factor (item) alone explained 57.72% of the total variance of the 8 items analyzed. Thus, the first 2 factors (items) explained 71.54% of the total eigenvalue variance. In Multimedia Appendix 4, we can see that the Cattell scree test validates Kaiser criteria because it is located between item 2 and item 3.

Examination of the factor structure of the initial scale revealed 2 factor axes (Table 1). When analyzing components 1 and 2 in relation to the 8 items before rotation, we observed a loading of all items for the first factor. We also observed 2 similar correlations between the 2 factors and item 7. We proceeded to a varimax rotation to obtain a simple factorial representation. After varimax rotation, we observed that items 1, 2, 3, 4, and 5 loaded on the first factor.

Item 1 corresponds to “I know how to find helpful health resources on the Internet,” item 2 corresponds to “I know how to use the Internet to answer my health questions,” item 3 refers to “I know what health resources are available on the Internet,” item 4 refers to “I know where to find helpful health resources on the Internet,” and item 5 corresponds to “I know how to use the health information I find on the Internet to help me*.*” For factor 2, items 6 and 7 were loaded. Item 6 corresponds to “I have the skills I need to evaluate the health resources I find on the Internet*”* and item 7 refers to “I can tell high quality from low quality health resources on the Internet.” Item 8, corresponding to “I feel confident in using information from the Internet to make health decisions,” seems to straddle the 2 factors after varimax rotation.

**Table 1:** Principal component factor analysis before and after varimax rotation^a,b^.

Item	Principal component analysis before varimax rotation		Principal component analysis after varimax rotation	
	Factor 1	Factor 2	Factor 1	Factor 2
1	*0.79* ^c^	−0.36	*0.86*	0.12
2	*0.86*	−0.31	*0.89*	0.21
3	*0.86*	−0.27	*0.87*	0.23
4	*0.80*	−0.13	*0.74*	0.32
5	*0.79*	0.03	0.65	0.45
6	0.66	0.58	0.24	*0.85*
7	0.61	0.66	0.16	*0.88*
8	0.66	0.13	0.48	0.46

^a^Variance accounted for=71.54%.

^b^Cronbach α=.89.

^c^Scores >0.7 have been italicized.

#### Patterns of Interitem Correlation

After analysis of the correlation matrix (Multimedia Appendix 5), we found that the 8 items in F-eHEALS are positively correlated with each other. The values were in the range of 0.30 to 0.86. The lowest correlation recorded was between item 1 and item 7 (*r*=0.3). In contrast, the highest correlation observed was between item 2 and item 3 (*r*=0.86). Item 7 appeared to have the lowest correlation with the other items. The average interitem correlations ranged from 0.53 to 0.78 (Table 2). The means of the interitem correlations of the original eHEALS scale ranged from *r*=0.51 to 0.76 [7], which is close to the F-eHEALS results.

**Table 2:** Descriptive statistics by items (N=328).

Item	n (%)	Mean (SD)^a^	Range	Interitem correlation	Original interitem correlation, (Norman and Skinner [7])
1	328 (100)	3.31 (1.06)	1-5	0.69	0.68
2	328 (100)	3.25 (1.14)	1-5	0.78	0.70
3	328 (100)	3.32 (1.11)	1-5	0.78	0.68
4	328 (100)	3.53 (1.06)	1-5	0.71	0.76
5	328 (100)	3.48 (1.07)	1-5	0.71	0.73
6	328 (100)	3.22 (1.19)	1-5	0.59	0.63
7	328 (100)	3.72 (1.08)	1-5	0.53	0.55
8	328 (100)	2.34 (1.03)	1-5	0.57	0.51

^a^Overall mean (SD)=26.16 (6.61).

#### Concurrent Validity of the Scale

The F-eHEALS score correlated positively and significantly with the HLS-EU–Q16 score (*r*=0.34; *P*<.001) and the PAM-13 score (*r*=0.31; *P*<.001). We did not observe significant difference between gender (*F*_3,324_=1.56; *P*=.20), health outcomes (chronic disease; *F*_1,326_=0.017; *P*=.89), and F-eHEALS score.

## Discussion

### Principal Findings

The objective of the study was to translate eHEALS to French and validate F-eHEALS in a student population. The results of this study validated its translation and adaptation, allowing us to propose a French version of the validated eHEALS scale (Multimedia Appendix 6).

### Comparison With Previous Studies

#### Translation

The translation process highlighted the complexity of translating from English to French. Although the translated content must remain the same as the original version, reflecting the real meaning of the items, it also has to be adapted to the language and culture of the target population. During the double reverse translation, terms were discussed by the expert committee—specifically, *health resources*, having been mentioned in other translations [38,45], illustrates the universal complexity of translating from English to other languages. After conducting the pretest measurement to check the clarity of the items, the scale was presented to a sample of 328 students.

#### Validation

#### Fidelity

The fidelity of F-eHEALS was measured using internal consistency and temporal stability. Internal consistency was evaluated using Cronbach α, and temporal stability was evaluated by confidence index. In our study, internal consistency (Cronbach α=.89) was judged to be excellent according to recommendations of Nunnaly [60]. Regarding temporal stability, we observed good fidelity [63] of our sample. These results are congruent with those of the original study [7], which obtained a similar result (Cronbach α=.88). Moreover, our results regarding fidelity are consistent with those of several studies that have shown a higher Cronbach α than the original (ie, >.88) [27-29,38-41].

#### Construct Validity

The Bartlett sphericity test was significant, and the KMO sampling precision index can be described as excellent. These results indicate that the correlations between the items are of good quality and thus legitimize the factor analysis. In addition, as the current sample includes 328 participants, this was correct and the statistical power was considered to be sufficient [61].

Construct validity highlighted a 2-factor (or 2D) structure. Although this contradicts some studies [27,28,33,35,40,41,64], other studies have also revealed a 2-factor structure [30,37,39,49,65-67]. This 2-dimensionality is fully consistent with the multidimensional property of eHealth literacy, which is composed of different literacies [6]. These results are consistent with 3 studies [65,67,68] that found the item structure similar to ours (ie, information seeking: items 1, 2, 3, 4, 5, and 8 and information appraisal: items 6 and 7). Nevertheless, in the systematic analyses, Lee et al [18] demonstrated the lack of high-quality evidence for structural validity and internal consistency for 2-factor scales in the 3 studies, which shows the instability of 2-factor scales. Therefore, it is important to remain cautious about this structure.

We observed a problematic load factor for item 8. In the item loading analysis, item 8 was in the first factor before rotation; however, item 8 straddles the 2 factors after varimax rotation. This analysis is consistent with the Italian translation and validation before rotation [65]. It is likely that if the authors had rotated, they would probably have found similar results. Many other similarities were observed between this study and the Italian validation study (eg, Cronbach α, variance accounted, and 2D scale). This is probably owing to the common Latin roots of French and Italian languages. However, these similarities are not observed in other Latin translations (eg, Portuguese and Spanish). Moreover, in an Italian validation with a population of nurses, De Caro et al [33] observed a unidimensionality. This shows the instability of eHEALS according to the population. Item 8 also seemed problematic in other validations observing a 2D scale [39]. This is likely owing to the fact that in the original article, the loading of item 8 was not excellent. Item 8 loads at 0.6 without rotation, which does not seem to be *good* [62]. However, to the best of our knowledge, no study on the validation of eHEALS has removed item 8 from the questionnaire, despite its weakness. Therefore, despite the difficulties of discrimination and the ambiguous load factor of item 8, consistent with Dale et al [39], Gazibara et al [37], Richtering et al [66], and Shiferaw [30], we decided to retain it.

Depending on the language, item 8 can switch between the first [37] and second factors [65]. Items 1 to 5 begin with “I know,” whereas items 6 and 7 refer to the notion of self-evaluation, such as “I have the skills” or “I can.” Item 8 seems to be close to the notion of reliability and trust (“I feel confident”) and, therefore, to the notion of self-evaluation including items 6 and 7. Thus, this double factor divides the items into two dimensions: those measuring information-seeking skills (items 1-5) and those measuring the evaluation of health information (items 6-8).

It would seem appropriate to measure a mean score for each underlying factor, rather than an overall score. However, considering the instability of some items, it seems more relevant to measure an overall score. Moreover, a score for each factor can compromise comparisons and standardization, with most studies using an overall score. Considering these indications, we suggest calculating scores for each factor and an overall score in future uses of F-eHEALS.

The variance explained by the 2-factor model in this study is also relatively high compared with that in other similar validation studies in other languages [27,32,35].

The quality of representation of the items (ie, whether the items are well represented by the dimensions of the construct) was judged to be excellent because all the items showed a score >0.45. Thus, we have decided to retain all items in the translation and validation of eHEALS. The means of the interitem correlations of the original eHEALS scale [7] are consistent with the F-eHEALS results. The effect of the F-eHEALS scale construction was acceptable. To validate its content, we formulated 3 hypotheses, which have proved to be correct. The first hypothesis, according to which there was no link between user characteristics (age, gender, and health status) and the level of eHealth literacy, was validated. We found no significant correlations between the F-eHEALS scores and the sociodemographic characteristics of our sample. These results are consistent with the results of other studies regarding gender [44,47] and health outcomes [44]. Our second hypothesis, according to which there was a link between health literacy and eHealth literacy, was validated. In addition, the health literacy score, measured using HLS-EU–Q16, was positively and moderately correlated with the F-eHEALS score. These results are consistent with those of other studies [38,45,48,49]. Our third hypothesis, expecting a link between the level of patients’ health activation and the level of eHealth literacy, was validated. The patient health activation score, measured using the PAM-13, was positively and moderately correlated with the F-eHEALS score, which is consistent with the study by Lee et al [51].

### Limitations

This study has some limitations. First, our sample was very homogenous in terms of important factors influencing eHealth literacy, as it was composed exclusively of young adult students for a practical reason. Moreover, this scale was administered to different categories of individuals, such as older adults [40], young adults [41], nursing students [42], and teenagers [43]. Therefore, this scale should be validated in a more representative sample of French-speaking populations in a subsequent study. Second, the PAM-13 scale has only been partially validated. Only the analysis of internal consistency and temporal stability was conducted [58]. A more complete validation is necessary. Similarly, confirmatory factor analysis should be performed. Third, as F-eHEALS is a 2D scale, we recommend scoring the 2 subscales separately and measuring the overall score, as done in this study. Fourth, French scientific literature is relatively scarce in the area of health literacy. Our comparisons were made on the basis of a wide variety of cultures and languages. We hope that translations of scales, such as F-eHEALS, will promote studies in the field. Fifth, eHEALS, HLS-EU–Q16, and PAM-13 are subjective assessments [38], as the level of eHealth literacy is self-reported by respondents and may be overestimated or underestimated. Therefore, it will be interesting to clarify the links between subjective and objective assessments to better understand the margins of error in these tests.

Recently, since the development of interactive applications—social networks, forums, and so on (Health 2.0)—that help people communicate about their health, eHealth literacy is required to be considered as a broader skill [35]. To bridge the *digital divide*, it will be important to use instruments that measure all varieties of eHealth literacy. Even if eHEALS is strongly correlated with DHLI [21], considering these aspects of interaction, we encourage French-speaking researchers to integrate new items related to these new forms of interaction into eHEALS or to translate and validate DHLI [21] to measure the variability of eHealth literacy encompassing the competencies from Health 2.0 and to allow the French-speaking community to catch up in this field.

### Conclusions

This study was conducted with a population of young adult students for a practical reason, which allowed us to propose the first eHealth literacy scale that is validated in terms of fidelity and the validity of F-eHEALS to the French-speaking community. This 2D scale will need to be generalized to other populations in a French-speaking context. Finally, a version considering interactive applications (ie, Health 2.0 and DHLI scale) should be considered on the basis of this study. The value of such a scale seems to be even more relevant as eHealth has never been as much in demand as in recent years and will probably be even more so in the future, particularly owing to the increasing use of eHealth technologies. We hope that this study will enable other authors to initiate studies in the field of eHealth literacy in the French context.

## Appendix

Multimedia Appendix 1 Steps described by Vallerand [54].

Multimedia Appendix 2 Presentation of the clarity of the items (and instructions) of the French version of eHealth Literacy Scale, judged by 22 volunteer laypeople.

Multimedia Appendix 3 Details of respondent characteristics (N=328)—sociodemographic characteristics, health status, health literacy level, and eHealth literacy level.

Multimedia Appendix 4 Explanation of variance using Cattell scree test on item-specific score values.

Multimedia Appendix 5 Interitem correlation matrix for the French version of eHealth Literacy Scale.

Multimedia Appendix 6 The French version of eHealth Literacy Scale.

## Abbreviations

DHLI: Digital Health Literacy Instrument
eHEALS: eHealth Literacy Scale
F-eHEALS: French version of eHealth Literacy Scale
HLS-EU–Q16: Health Literacy Survey-Europe–16
KMO: Kaiser-Meyer-Olkin
PAM-13: Patient Activation Measure–13
