# Supplementary figures and images for: Translation and Validation Study of the French Version of the eHealth Literacy Scale: Web-Based Survey on a Student Population
Source: JMIR Form Res. 2022 Aug 31;6(8):e36777. doi: 10.2196/36777 (PMC9475413; doi:10.2196/36777)

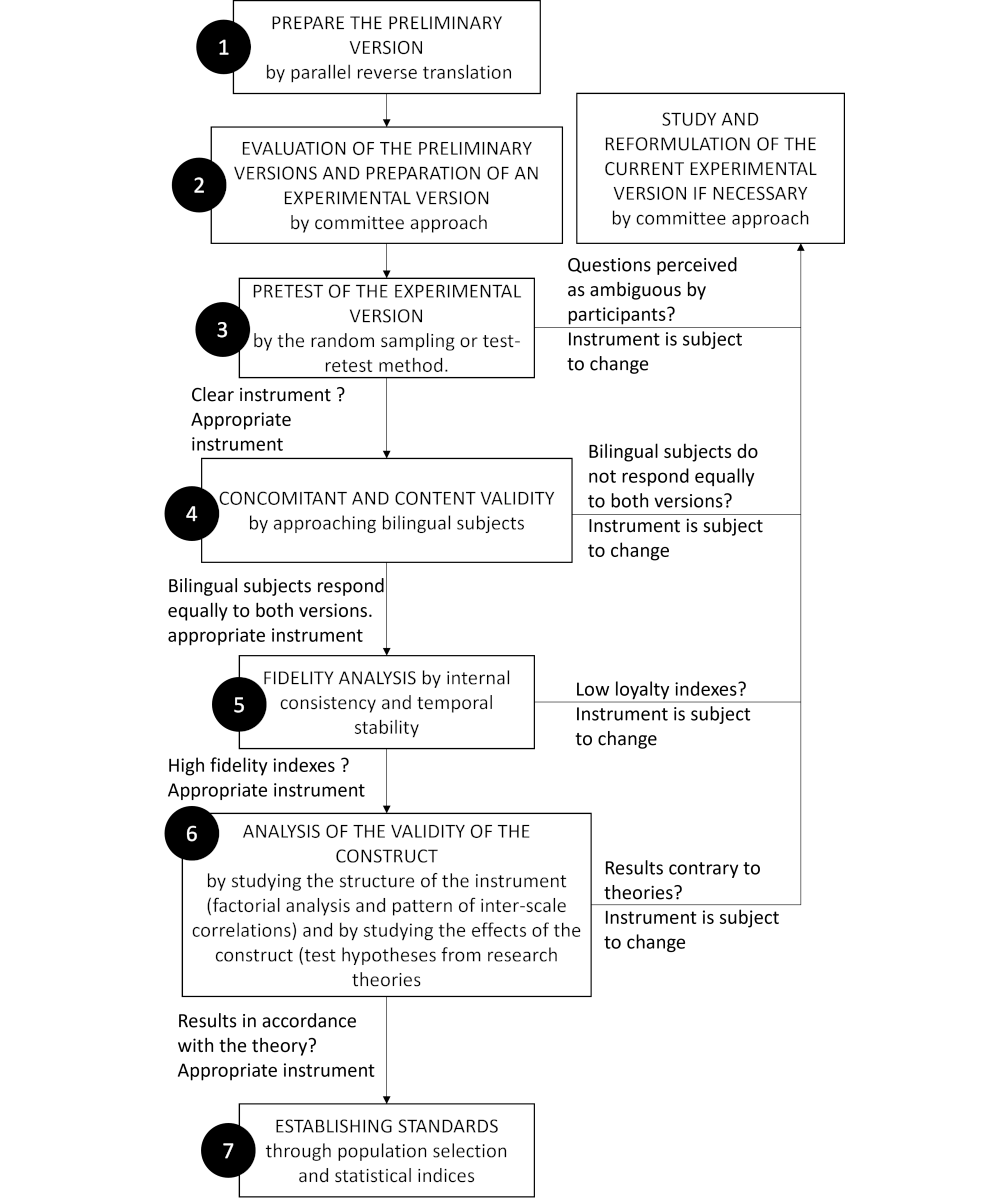

Supplement: Multimedia Appendix 1 [file formative_v6i8e36777_app1.png]

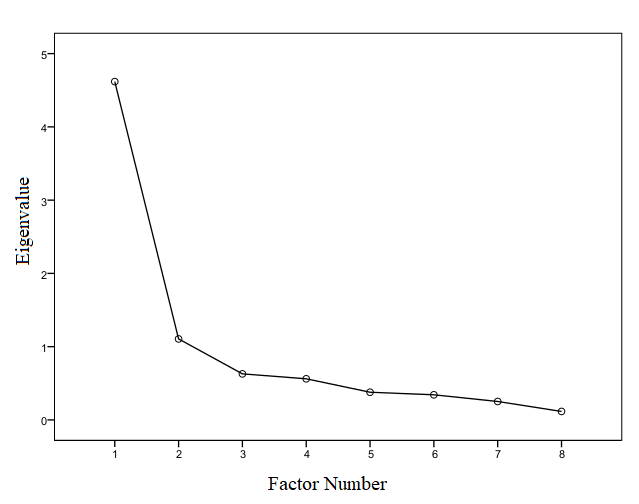

Supplement: Multimedia Appendix 4 [file formative_v6i8e36777_app4.png]
