# Supplementary material for: Translation and Validation Study of the French Version of the eHealth Literacy Scale: Web-Based Survey on a Student Population
Source: JMIR Form Res. 2022 Aug 31;6(8):e36777. doi: 10.2196/36777 (PMC9475413; doi:10.2196/36777)
Supplement: Multimedia Appendix 2 [file formative_v6i8e36777_app2.docx]

Annexe 2

Table : Presentation of the clarity of the items (as well as the instructions) of the French version of eHEALS, judged by 22 volunteer laypeople.

| **Items (N = 22)** | **Moyenne** | **Ecart-Type** |
| --- | --- | --- |
| Instruction: **I would like to ask you for your opinion and about your experience using the Internet for health information. For each statement, tell me which response best reflects your opinion and experience *right now*.** | 6.45 | 0.74 |
| *supplementary item* 1 : How **useful** do you feel the Internet is in helping you in making decisions about your health? | 6.68 | 0.47 |
| *supplementary item* 2 : How **important** is it for you to be able to access health resources on the Internet? | 6.27 | 1.24 |
| Item 1 : I know how to find helpful health resources on the Internet | 5.59 | 2.04 |
| Item 2 : I know how to use the Internet to answer my health questions | 6.05 | 1.59 |
| Item 3 : I know what health resources are available on the Internet | 6.05 | 1.21 |
| Item 4 : I know where to find helpful health resources on the Internet | 6.64 | 0.79 |
| Item 5 : I know how to use the health information I find on the Internet to help me | 6.36 | 1 |
| Item 6 : I have the skills I need to evaluate the health resources I find on the Internet | 6 | 1.41 |
| Item 7 : I can tell high quality from low quality health resources on the Internet | 6.41 | 1.1 |
| Item 8 : I feel confident in using information from the Internet to make health decisions | 6.45 | 1.06 |
| Total | 6.27 | 1.15 |
