# Supplementary material for: Translation and Validation Study of the French Version of the eHealth Literacy Scale: Web-Based Survey on a Student Population
Source: JMIR Form Res. 2022 Aug 31;6(8):e36777. doi: 10.2196/36777 (PMC9475413; doi:10.2196/36777)
Supplement: Multimedia Appendix 3 [file formative_v6i8e36777_app3.docx]

Appendix 3

Table: Details of respondent characteristics (n = 328): socio-demographic characteristics, health status, health literacy level and e-health literacy level.

| **Respondent characteristics (n = 328)** | | **Effectifs (%)** |
| --- | --- | --- |
| Sociodemographic characteristics | | |
| Age Mean 21.22 years (SD = 2.7, range = 16-33) | | |
| Gender group (M/F) | Female  Male  Others | 274 (83.5)  52 (15.9)  2 (0.6) |
| Educational Level | First year Bachelor  Second year Bachelor  Third year Bachelor  First year Master  Second year Master  First year PhD  Second year PhD  Third year PhD | 58 (17.7)  65 (19.8)  123 (37.5)  53 (16.2)  20 (6.1)  5 (1.5)  2 (0.6)  2 (0.6) |
| Field of education | Arts  Law and Political Science  Economy-Management  Literature and languages  Medicine  Sciences  Engineering Sciences  Human and Social Sciences  Sport | 10 (3)  13 (4)  11 (3.4)  32 (9.8)  27 (8.2)  32 (9.8)  4 (1.2)  186 (56.7)  13 (4) |
| Health outcomes | | |
| With a chronic disease | Yes  No | 27 (8.2)  301 (91.8) |
| Additional scales : Patient Activation Measure (PAM-13) and Health Literacy Scale (HLS-EU-Q16) | | |
| PAM-13 | Mean =46.18/65 ; SD = 8.42 ; range = 13-65 | |
| HSL-EU-Q16 | Mean = 11.48/16. SD = 3.12 ; range = 0-16 | |
| e-health literacy scale (F-eHEALS) | | |
| F-eHEALS (8 items) | Mean = 26.16/40 ; SD = 6.6 ; range = 8-40 | |
