# Supplementary material for: Translation and Validation Study of the French Version of the eHealth Literacy Scale: Web-Based Survey on a Student Population
Source: JMIR Form Res. 2022 Aug 31;6(8):e36777. doi: 10.2196/36777 (PMC9475413; doi:10.2196/36777)
Supplement: Multimedia Appendix 5 [file formative_v6i8e36777_app5.docx]

Appendix 5: F-eHEALS inter-item correlation matrix on N = 328 participants

|  | Item 1 | Item 2 | Item 3 | Item 4 | Item 5 | Item 6 | Item 7 | Item 8 |
| --- | --- | --- | --- | --- | --- | --- | --- | --- |
| Item 1 | - |  |  |  |  |  |  |  |
| Item 2 | 0.77 | - |  |  |  |  |  |  |
| Item 3 | 0.69 | 0.86 | - |  |  |  |  |  |
| Item 4 | 0.56 | 0.61 | 0.68 | - |  |  |  |  |
| Item 5 | 0.57 | 0.57 | 0.58 | 0.67 | - |  |  |  |
| Item 6 | 0.33 | 0.42 | 0.45 | 0.42 | 0.51 | - |  |  |
| Item 7 | 0.3 | 0.37 | 0.37 | 0.36 | 0.42 | 0.65 | - |  |
| Item 8 | 0.44 | 0.49 | 0.44 | 0.46 | 0.46 | 0.38 | 0.41 | - |
