# Supplementary material for: Translation and Validation Study of the French Version of the eHealth Literacy Scale: Web-Based Survey on a Student Population
Source: JMIR Form Res. 2022 Aug 31;6(8):e36777. doi: 10.2196/36777 (PMC9475413; doi:10.2196/36777)
Supplement: Multimedia Appendix 6 [file formative_v6i8e36777_app6.docx]

Annexe 6

Evaluation de la clarté des items du questionnaire eHEALS

Nous souhaiterions recueillir votre opinion et votre ressenti par rapport à votre utilisation d’Internet comme outil pour obtenir des informations relatives à la santé. Pour chaque affirmation, indiquez la réponse qui reflète le mieux votre opinion et votre expérience en ce moment.

| 1. Dans quelle mesure pensez-vous qu’Internet vous est **utile** pour prendre des décisions concernant votre santé ?  - Pas du tout utile - Plutôt inutile - Ni utile, ni inutile - Plutôt utile - Très utile |
| --- |

| 1. Dans quelle mesure est-il **important** pour vous d'avoir accès à des sources d’information sur la santé sur Internet ?  - Pas du tout important - Plutôt pas important - Ni important, ni pas important - Plutôt important - Très important |
| --- |

| 1. Je sais **quelles** sources d’information sur la santé sont disponibles sur Internet.  - Pas du tout d'accord - Plutôt pas d'accord - Ni d'accord, ni pas d'accord - Plutôt d'accord - Tout à fait d'accord |
| --- |

| 1. Je sais **où** trouver des sources d’information utiles sur la santé sur Internet.  - Pas du tout d'accord - Plutôt pas d'accord - Ni d'accord, ni pas d'accord - Plutôt d'accord - Tout à fait d’accord |
| --- |

| 1. Je sais **comment** trouver des sources d’information utiles sur la santé sur Internet.  - Pas du tout d'accord - Plutôt pas d'accord - Ni d'accord, ni pas d'accord - Plutôt d'accord - Tout à fait d'accord |
| --- |

| 1. Je sais **comment utiliser** Internet pour trouver des réponses à mes questions sur la santé.  - Pas du tout d'accord - Plutôt pas d'accord - Ni d'accord, ni pas d'accord - Plutôt d'accord - Tout à fait d’accord |
| --- |

| 1. Je sais comment utiliser **les informations sur la santé** que je trouve sur Internet pour m’aider.  - Pas du tout d'accord - Plutôt pas d'accord - Ni d'accord, ni pas d'accord - Plutôt d'accord - Tout à fait d'accord |
| --- |

| 1. J'ai les compétences dont j'ai besoin pour **évaluer** les sources d’information sur la santé que je trouve sur Internet.  - Pas du tout d'accord - Plutôt pas d'accord - Ni d'accord, ni pas d'accord - Plutôt d'accord - Tout à fait d’accord |
| --- |

| 1. Je peux faire la différence entre les sources d’information sur la santé de **bonne** **qualité** et celles de **mauvaise qualité** sur Internet.  - Pas du tout d'accord - Plutôt pas d'accord - Ni d'accord, ni pas d'accord - Plutôt d'accord - Tout à fait d’accord |
| --- |

| 1. J'ai **confiance** en l'utilisation des informations provenant d'Internet pour prendre des décisions en matière de santé.  - Pas du tout d'accord - Plutôt pas d'accord - Ni d'accord, ni pas d'accord - Plutôt d'accord - Tout à fait d’accord |
| --- |
